# Spatio-Temporal Analysis of the Accuracy of Tropical Multisatellite Precipitation Analysis 3B42 Precipitation Data in Mid-High Latitudes of China

**DOI:** 10.1371/journal.pone.0120026

**Published:** 2015-04-01

**Authors:** Yancong Cai, Changjie Jin, Anzhi Wang, Dexin Guan, Jiabing Wu, Fenghui Yuan, Leilei Xu

**Affiliations:** 1 State Key Laboratory of Forest and Soil Ecology, Institute of Applied Ecology, Chinese Academy of Sciences, Shenyang, Liaoning, People’s Republic of China; 2 Graduate University of Chinese Academy of Sciences, Beijing, People’s Republic of China; 3 Institute of Scientific and Technical Information, Chinese Academy of Tropical Agricultural Sciences, Danzhou, Hainan, People’s Republic of China; University of California San Diego, UNITED STATES

## Abstract

Satellite-based precipitation data have contributed greatly to quantitatively forecasting precipitation, and provides a potential alternative source for precipitation data allowing researchers to better understand patterns of precipitation over ungauged basins. However, the absence of calibration satellite data creates considerable uncertainties for The Tropical Rainfall Measuring Mission (TRMM) Multisatellite Precipitation Analysis (TMPA) 3B42 product over high latitude areas beyond the TRMM satellites latitude band (38°NS). This study attempts to statistically assess TMPA V7 data over the region beyond 40°NS using data obtained from numerous weather stations in 1998–2012. Comparative analysis at three timescales (daily, monthly and annual scale) indicates that adoption of a monthly adjustment significantly improved correlation at a larger timescale increasing from 0.63 to 0.95; TMPA data always exhibits a slight overestimation that is most serious at a daily scale (the absolute bias is 103.54%). Moreover, the performance of TMPA data varies across all seasons. Generally, TMPA data performs best in summer, but worst in winter, which is likely to be associated with the effects of snow/ice-covered surfaces and shortcomings of precipitation retrieval algorithms. Temporal and spatial analysis of accuracy indices suggest that the performance of TMPA data has gradually improved and has benefited from upgrades; the data are more reliable in humid areas than in arid regions. Special attention should be paid to its application in arid areas and in winter with poor scores of accuracy indices. Also, it is clear that the calibration can significantly improve precipitation estimates, the overestimation by TMPA in TRMM-covered area is about a third as much as that in no-TRMM area for monthly and annual precipitation. The systematic evaluation of TMPA over mid-high latitudes provides a broader understanding of satellite-based precipitation estimates, and these data are important for the rational application of TMPA methods in climatic and hydrological research.

## Introduction

Precipitation is a key variable for the Earth’s water cycle and energy balance, also plays a major role in monitoring water-related natural hazards and water resource management. Currently, many global climate models have predicted that climate change will alter the spatial patterns of precipitation at a global scale and have showed a general change will occur in the timing and amount of a given daily precipitation [1–3]. Precipitation exerts major effects on the earth’s ecosystem [4] and hydrological cycle. Accurate measurement of precipitation is essential to investigate spatial pattern of rainfall at regional scale. Having accurate rainfall data will improve our understanding of the effect of precipitation on hydrology and climate change. Traditionally, rain gauge is a main or even the only means of obtaining detailed rainfall data. However, the limitations of rain gauge measurement restrict our understanding of precipitation: one is the insufficient spatial representation [5], which means that direct measurements of rainfall at an single station were generally not very useful in making estimates of areal rain and spatial distribution of rainfall over large areas; the other is sparse distribution over mountainous areas and unavailability over the oceans [6, 7]. A long history of the development in the estimation of precipitation data based on satellite data has culminated in sophisticated satellite instruments and techniques that can be used to combine information from multiple satellites to produce long time series products that are useful for climate monitoring [8].

Currently, many operational satellite-based precipitation products have been available at a global scale, e.g., TMPA 3B42 [9], Precipitation Estimation from Remotely Sensed Information Using Neural Networks [10, 11], the Climate Prediction Center Morphing Method [12]. These products have potential application in climate change and hydrological models, as well as rain regime and weather forecasting. However, the resulting precipitation estimates suffered from various types of errors, namely: non-negligible bias, random errors associated with inadequate sampling, algorithm errors, and the indirect nature of the physical relationship between precipitation and the observations [6, 13]. These errors associated with precipitation tend to propagate in climate or hydrological models, and lead to convey some misleading information for decision-makers. Hence, the evaluation of precipitation estimates is a prerequisite work to its utilization in practical applications.

This study focuses on the evaluation of TMPA, a quasi-global precipitation product. A wide range of studies have evaluated TMPA worldwide, such as studies in Asia [5, 14, 15], South America [16–19], North America [20, 21], Europe [22], Africa [23]. Generally, two main methods have been employed to evaluate the accuracy of satellite-based precipitation estimates: a direct comparison of rain gauge data and satellite data [5, 15, 17, 24–26], and an indirect analysis of derivation variable outputting from models, e.g., hydrological [27–31] and crop yield models [32] that are driven by precipitation data. However, most numerical models are rife with sources of uncertainty [33–35]. When the latter method is adopted to assess satellite-based precipitation data, this inevitably results in much more biases and further reduced reliability. Therefore, an analysis that directly compares various precipitation data would be an effective method that should provide a reasonable evaluation of quality of precipitation data. When these studies related to the evaluation of TMPA were reviewed, their performances were inconsistent and varied from place to place. In addition, numerous previous studies focused on low latitudes regions within 40°N-S, to our best knowledge, few studies have been conducted in mid-high latitudes regions [27]. Hence, work in mid-high latitudes regions is urgently needed to provide a comprehensive insight into the accuracy of TMPA and allow its extensive application in science community.

The main objective of our study is to assess the accuracy of TMPA 3B42 V7 (hereinafter referred to as TMPA V7) data over the mid-high latitudes region of China. This region is situated beyond the nominal coverage (38°NS) of the TRMM Microwave Imager (TMI) and Precipitation Radar (PR) [36]. As Huffman et al. [9] discussed, the TRMM Combined Instrument (TCI) that combines data from TMI and PR is an important data source that can be used to calibrate the two main input data sources, including microwave and infrared satellite observations, for processing a TMPA product. Because these necessary calibration data are lacking beyond 40°NS, the calibration coefficients for low latitudes regions must be used to this special region [9]. Obviously, large biases and errors would exist over this region. In addition, various climate zones characterize this region. Therefore, northern China serves as a unique place that can provide an opportunity to assess the accuracy of TMPA under different climatic conditions and geographical features. First, this study adopts some common accuracy indices to provide a quantitative description of TMPA V7 in terms of error in precipitation amount and in detecting the occurrence of precipitation events. Then, the accuracy of TMPA at multiple timescales (daily, monthly and annual scale) is assessed separately. In addition, spatial and temporal trends in the accuracy indices are also examined to show how the performance of TMPA changes over time and space.

## Materials and Methods

### Study area

**Fig 1:**
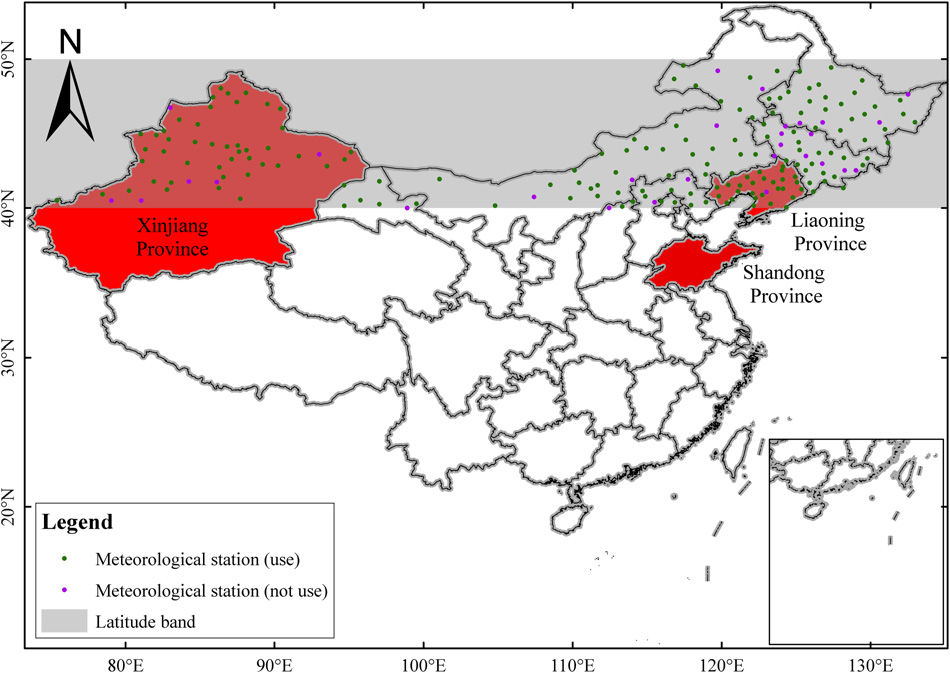
Geographical location of study area within China and the spatial distribution of meteorological station. The study area lies within the shaded region between 40° and 50°N. Also, three regions are filled with red.

**Fig 2:**
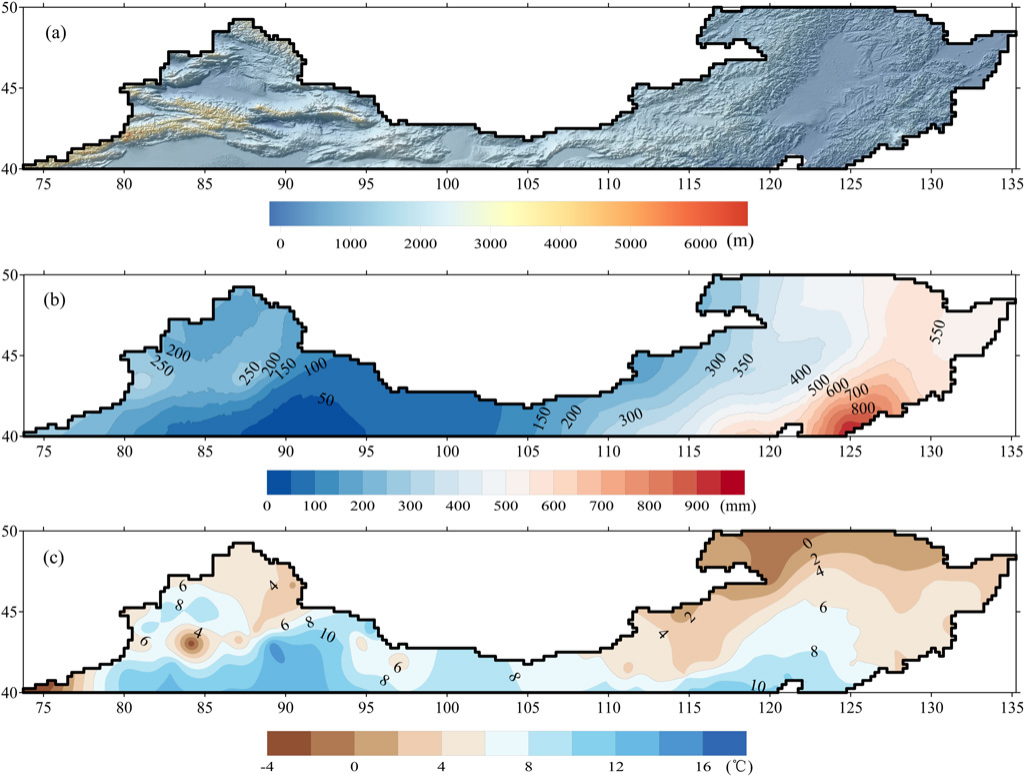
The spatial distribution of terrain and elevation, mean annual precipitation and mean air temperature during 1990–2012 across the study area: (a) terrain and elevation, (b) precipitation and (c) air temperature. Shuttle Radar Topography Mission (SRTM) DEM with spatial resulution of 90 meters is resampled to 0.02° to represent terrain. Kriging method is used to interpolate observations from 169 weather stations during 1990–2012 to maps of precipitation and temperature.

Fig. 1 shows the longitudinal region between 40 and 50°N. This region spans from northeast to northwest China in the mid-high latitudes ranging from 73.25°E to 135.25°E. The topography varies remarkably in the study area, forming a more complex terrain in the western part than the eastern part of this region (Fig. 2A). Mountains dominate in the western part with a mean elevation above 3000 m, while the eastern part features flat plains. Most precipitation falls as snow with the typical low temperatures over this region from the end of October to March in next year. The rainy season normally lasts 4 months stretching from June through September. However, the precipitation and temperature vary spatially and significantly in this region (Fig. 2B and C). Generally, precipitation increases from northeast to northwest between 0 and 1000 mm annually, and mean annual temperature decreases from south (16°C) to north (−4°C). In addition, this region is characterized by a mixed climate zone, from a humid region in the northeastern part to an arid region in the northwestern part.

### Data source

The new version TMPA V7 data, after retrospective reprocessing, released in December 2012, is available with a spatial resolution of 0.25° and a temporal resolution of 3 h within 50°N and 50°S global latitude from 1998 to present. This product has undergone several updates, including incorporating additional satellite observation data, improved algorithms, and adopting newly advanced gauge analysis [21]. Chen et al. [37] provided detailed information on TMPA V7. To be temporally consistent with the daily observation data (20:00-20:00 UTC+8), the TMPA 3 h precipitation products have been accumulated into daily precipitation estimates starting from 12:00 UTC in a previous day to 12:00 UTC in a current day. To compute the daily rainfall from the TMPA rainfall rate, the rainfall rates at 12:00 UTC (previous day) and 12:00 UTC (current day) were accumulated for 1.5 h while rainfall at other times (i.e., 15:00, 18:00, 21:00, 00:00, 03:00, 06:00, and 09:00 UTC) were accumulated for 3 h. Then, satellite-based precipitation estimates at monthly and annual timescales are derived from daily TMPA V7 by a simple accumulation.

**Fig 3:**
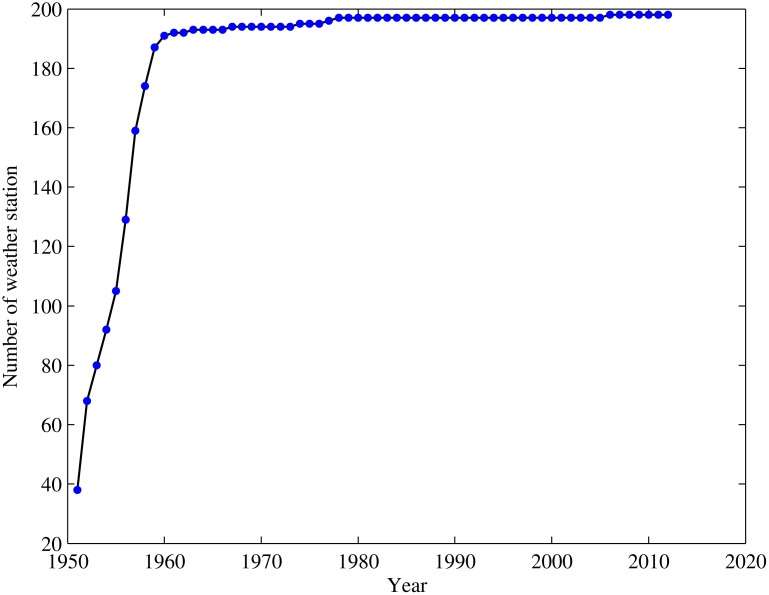
Number of available weather stations within the study area during the period of 1950–2012. Each dot represents the number of weather stations with complete data records for use during analysis for one year.

In this region, the observed precipitation dataset, prepared by the National Weather Department of China, is consist of 198 weather stations during 1951–2012. Its spatial distribution is rather homogeneous in the territory. Nevertheless, different stations have available time series of data of different lengths (Fig. 3). Because some rain gauge measurement sites lie on the boundary of TRMM grid box, determining which grid box a particular gauge belongs proved difficult, this may increase error and uncertainty when making a comparison based on grid-point. After excluding these gauges, the remaining 169 rain gauges that lie clearly within a single grid box were selected for the comparative analysis discussed below (Fig. 1). Then the observed monthly and annual precipitation time series were constructed based on daily data.

### Method

To present a quantitative evaluation of the accuracy of TMPA V7 data, a combination of continuous [38] and categorical statistical indices [39] were employed to assess the differences in precipitation amounts between TMPA and rain gauge and the ability of detecting occurrence of precipitation events. Continuous statistical indices consist of correlation coefficient (*CC*), mean error (*ME*) and mean absolute error (*MAE*). *CC* is a good measure of the degree of agreement between the two data sequences, that is, rain gauge observations and satellite-based precipitation data. *ME* and *MAE* were used to assess the average difference between the observed and satellite-based precipitation and the average magnitude of the error, respectively. These indices are defined as followings:

$$CC=\frac{\sum_{i=1}^{n}\left(Obs_{i}-\bar{Obs}\right)(Sat_{i}-\bar{Sat})}{\sqrt{\sum_{i=1}^{n}{\left(Obs_{i}-\bar{Obs}\right)}^{2}}•\sqrt{\sum_{i=1}^{n}{\left(Sat_{i}-\bar{Sat}\right)}^{2}}}$$ (1)

$$\begin{array}{c} ME=\frac{1}{n}\sum_{i=1}^{n}\left(Sat_{i}-Obs_{i}\right) \end{array}$$ (2)

$$\begin{array}{c} MAE=\frac{1}{n}\sum_{i=1}^{n}\left|Obs_{i}-Sat_{i}\right| \end{array}$$ (3)

where, *Obs*_*i*_ and *Sat*_*i*_ is the *i*th of time series of precipitation obtained from rain gauge and satellite, respectively; $\bar{Obs}$ and $\bar{Sat}$ denotes the mean of precipitation time series for the rain guage and satellite, respectively; *n* is the total number of observed and satellite-based precipitation data pairs.

Three widespread categorical indices, including probability of detection (*POD*), probability of false detection (*POFD*) and equitable threat score (*ETS*), were used to assess the skill in detection of precipitation events [39]. *POD* describes what fraction of the observed rainy events were correctly forecasted. *POFD* is the fraction of the observed no rainy events were incorrectly forecasted as rainy events. Both *POD* and *POFD* range from 0 to 1, with 1 being a perfect *POD* and 0 being a perfect *POFD*. *ETS* measures how well the rainy days estimated from satellite data can correspond to the observed rainy events, accounting for hits due to chance, and ranges from a poor score ($-\frac{1}{3}$) to a best score (1). The numerical weather prediction community commonly use *ETS* as an overall skill measure, whereas *POD* and *POFD* provide complementary information related to false detections and hits. Noted that, as suggested in many previous studies [27, 40–42], the common threshold of 1.0 mm/day is adopted to compute three categorical indices, shown in the following Equations.

$$\begin{array}{c} POD=\frac{h}{h+m} \end{array}$$ (4)

$$\begin{array}{c} POFD=\frac{f}{f+c} \end{array}$$ (5)

$$\begin{array}{c} ETS=\frac{h-r}{h+f+m-r},r=\frac{\left(h+m)(h+f\right)}{n} \end{array}$$ (6)

where, *h* is the number of hits cases where observed rain was correctly detected by satellite; *m* is the number of misses cases where observed rain was not detected; *f* is the number of false alarms cases where rain was detected but not observed on the ground; *c* is the number of correction cases where no rain was observed nor detected by satellite.

## Results

### Overall comparison of the TMPA product against rain gauge observations at various scales

#### Comparative analysis at daily, monthly and annual scales

**Fig 4:**
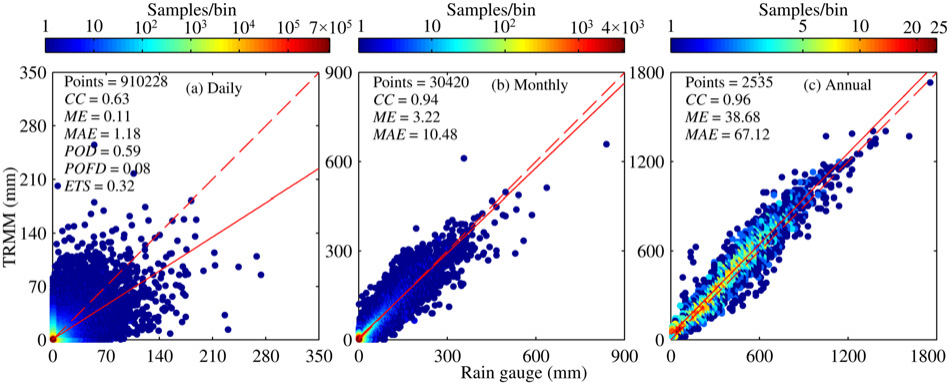
Density scatter plots of Tropical Multisatellite Precipitation Analysis (TMPA) versus rain gauge at three time scales: (a) Daily, (b) Monthly, (c) Annual. The 1:1 line of perfect agreement (red dashed line) and the linear fit line (red solid line) are indicated on each plot. Some statistics computed are also given on each plot. Note: correlation coefficient (*CC*), mean error (*ME*), mean absolute error (*MAE*), probability of detection (*POD*), probability of false detection (*POFD*), and equitable threat score (*ETS*).

An overall comparison of daily precipitation between TMPA V7 and rain gauge during 1998–2012 is described in Fig. 4A. A moderate correlation was observed between TMPA V7 and rain gauge with a *CC* of 0.63, indicating that these two datasets are in good agreement to some degree. This finding is comparable to or even better than that observed in low latitudes regions [19, 43]. Results of *ME* give an indication of a slight overestimation of daily precipitation, about 0.11 mm, by TMPA during 1998–2012. Moreover, the absolute error between the two precipitation datasets is 1.18 mm on average over this region. Table 1 lists some basic statistics (Max, Min, Mean, Sd, Rbias, and Abias) related to daily precipitation timeseries, it is indicated that there are some minor differences between two data sources. In general, bias is not so large that TMPA V7 can reproduce daily precipitation well. Moreover, three categorical indices also have acceptable scores. About 59% rainy events can be detected correctly by TMPA V7 among all the observed rainy events. Yet, satellites still falsely detect a certain rain days, so, especially, it may be difficult to make safe and reasonable decision related to reservoir regulation over regions with high density of river during rainy season. Overall, TMPA V7 shows a desirable skill in detecting no rain or rain event with *ETS* of approximately 0.32.

**Table 1:** Summary of basic statistics for TMPA and rain gauge at different timescales.

–	(Rain Gauge, TMPA)				–	
Timescale	Max (mm)	Min (mm)	Mean (mm)	Sd (mm)	Rbias (%)	Abias (%)
Day	(317.70,255.14)	(0,0)	(1.07,1.15)	(5.03,4.91)	7.48	106.54
Month	(840.80,659.78)	(0,0)	(32.11,34.64)	(49.15,49.35)	7.88	31.64
Year	(1765.50,1742.25)	(3.40,9.99)	(385.31,415.66)	(245.07,254.71)	7.87	16.63
Spring	(116.90,107.32)	(0,0)	(0.74,0.79)	(3.18,3.30)	6.76	118.92
Summer	(317.70,255.14)	(0,0)	(2.54,2.70)	(8.55,8.11)	6.30	98.03
Autumn	(230.70,123.67)	(0,0)	(0.78,0.84)	(3.54,3.66)	8.97	108.97
Winter	(43.90,60.03)	(0,0)	(0.16,0.23)	(0.89,1.28)	43.75	187.5

Note: Max, Min, Mean, and Sd represent maximum, minimum, mean value and standard deviation of precipitation time series at various timescales for Tropical Multisatellite Precipitation Analysis (TMPA) and rain gauge, respectively. Rbias is the relative bias, which is the ratio of *ME* to the mean value of time series from rain gauge at respective timescale; Abias is the absolute bias, which represents the ratio of *MAE* to the mean value of time series from rain gauge at respective timescale.

Compared to result based on daily precipitation, some changes in the values of statistical indices were observed at monthly and annual scales (Fig. 4B and C). Clearly, correlation between TMPA and rain gauge has improved greatly, with *CC* increasing from 0.63 at daily scale to a value greater than 0.94 at monthly and annual scales. That is, TMPA V7 data are more consistent with the observed precipitation at a larger timescale. In terms of the numerical difference between two data sources analyzed here, two statistical indices, *ME* and *MAE*, also show a significant upward trend, meaning that the difference in precipitation between satellite and rain gauge increases with a longer timescale. Because monthly and annual time series are constructed by accumulating daily data, errors inherent in daily data could directly propagate to such derivatives and cause a cumulative effect. In fact, judging from Rbias and Abias listed in Table 1, the degree of overestimation of precipitation is not intensified by TMPA with similar Rbias and lower Abias at a larger scale.

**Fig 5:**
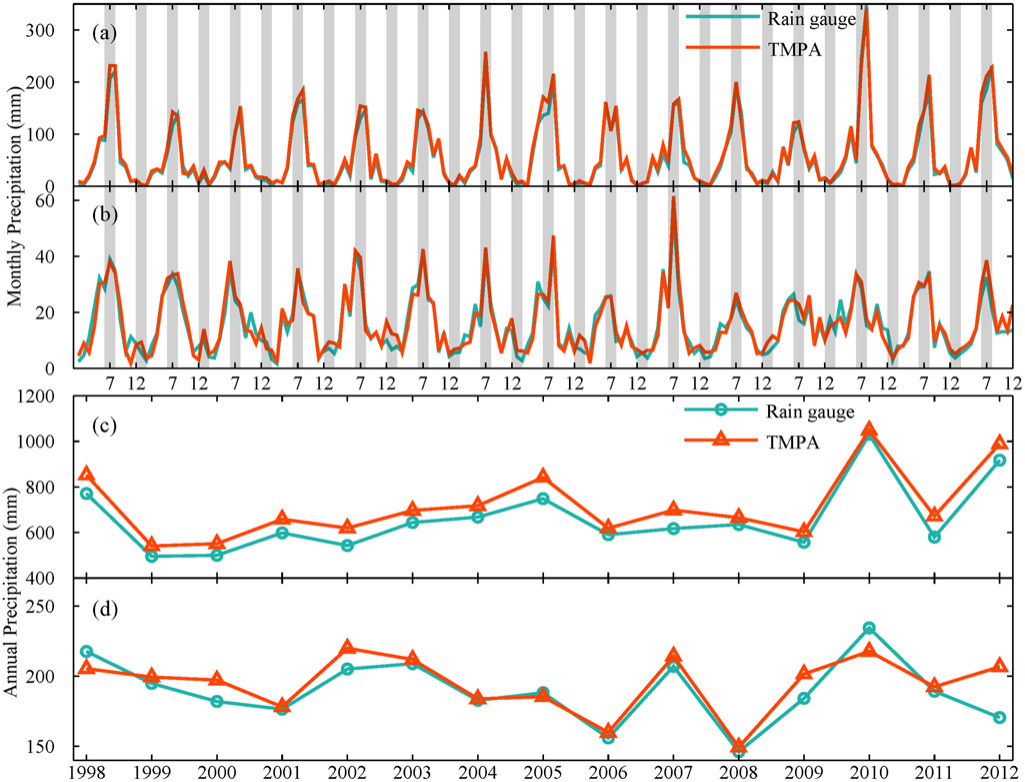
Variation of precipitation data from rain gauges and TMPA averaged over Liaoning ((a) and (c)) and Xinjiang province ((b) and (d)). Monthly and annual precipitation are shown in the first two panels and last two panels, respectively.

For a detailed description of variation in precipitation data collected from rain gauge and TMPA sources, two typical climatic regions were chosen for comparative analysis at regional scale (Fig. 5). These two regions were Liaoning (a humid area) and Xinjiang (an arid area) province, that experience significant differences in the amounts of precipitation (Fig. 2B and Table 2). Generally, precipitation estimate based on TMPA exhibits a high level of correspondence with the observation at monthly and annual scales. The *ME* for these two regions are all larger than 0, indicating that TMPA overestimates precipitation for both the humid and arid region (Table 3). This slight overestimation was 8.9% for Liaoning and 2.8% for Xinjiang. Thus, TMPA are not greatly affected by climatic conditions and could capture seasonal cycles and interannual variations in precipitation.

**Table 2:** Details description of three typical regions.

Region	Numbers of station	Elevation (m)	Temperature (°C)	Annual precipitation (mm)
Liaoning	27	251.26	8.44	657.85
Xinjiang	45	1351.07	7.87	153.97
Shandong	16	91.93	13.48	695.91

**Table 3:** Summary information for monthly and annual precipitation for five regions.

–	Month (mm)		Year (mm)	
Region	Mean*	*ME*	Mean*	*ME*
Liaoning	(54.97,59.84)	4.87	(659.62,718.13)	58.51
Xinjiang	(15.79,16.24)	0.45	(189.53,194.85)	5.32
Shandong	(59.10,60.75)	1.65	(709.19,729.01)	19.82
20-40 region	(83.77,87.17)	3.40	(1005.21,1046.00)	40.79
40-50 region	(33.00,36.23)	3.22	(396.04,434.72)	38.68

*: the format is a combination of data from Rain gauge and TMPA, e.g., (Rain gauge, TMPA).

#### Seasonal analysis of accuracy indices

**Fig 6:**
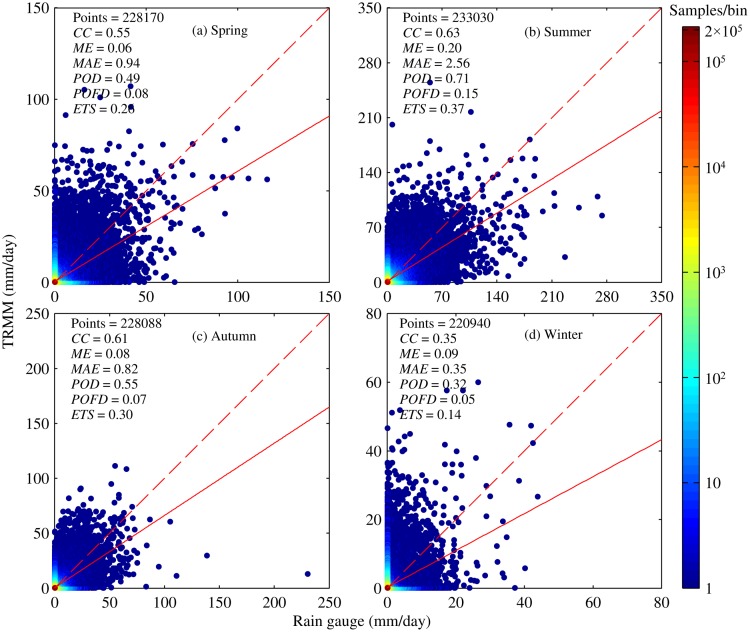
Density scatter plots of TMPA versus rain gauge at daily scale for four seasons: (a) Spring, (b) Summer, (c) Autumn, (d) Winter. The definitions and acronyms presented in each plot are the same as those used in Fig. 4.

The seasonal characteristics of error were investigated across this region (Fig. 6). Four seasons were defined as followings: spring, March through May; summer, June through August; autumn, September through November; and winter, December through February. Statistical indices did not have similar scores for all four seasons (Fig. 6). The best *CC* score appeared in summer (about 0.63), but in winter it had the lowest score (about 0.35). Except for spring, a moderate but good linear relationship was observed between TMPA V7 and rain gauge for each season over the mid-high latitudes regions with *CC* greater than 0.55.

To assess the difference in the amount of precipitation (Fig. 6), *ME* and *MAE* were calculated based on daily data for respective season. These two indices do not give similar patterns among the four seasons, with *ME* ranging from 0.06 to 0.20 mm and *MAE* ranging from 0.35 to 2.56 mm. The largest value for these two indices appeared in summer. Across this region, the rainy season occurs in summer, accounting for more than 60% of total annual precipitation. However, due to limited temporal sampling of satellite sensors, it has a large possibility of missing some rainy events characterized as short duration and high intensity so that larger errors were expected in summer. Even though small errors appear in winter, relative and absolute biases during the winter are largest among seasons, 43.75% and 187.5%, respectively (see Table 1). Obviously, overestimation of precipitation by TMPA was observed to be weak for the other three seasons, with smaller relative and absolute bias.

In terms of skill in detection of precipitation events, TMPA still does not have similar performance among seasons (Fig. 6). Summer had the best *POD* score, and the lowest one appears in winter again. This indicates that TMPA data are more useful in capturing rain events than snowfall. However, the score of *POFD* did not differ significantly among seasons, ranging from 0.08 to 0.15. For each season, TMPA data gave a similar false detection rate of precipitation events, so *POFD* appears to be season-independent. Result of *ETS* was similar to that of *POD*. Therefore, it is found that TMPA can be used most effectively to detect precipitation events with high scores in summer. This provides some evidences for the potential application of TMPA in a certain areas with insufficient historical observations from rain gauges, especially in the analysis of the characteristics of precipitation during summer.

**Fig 7:**
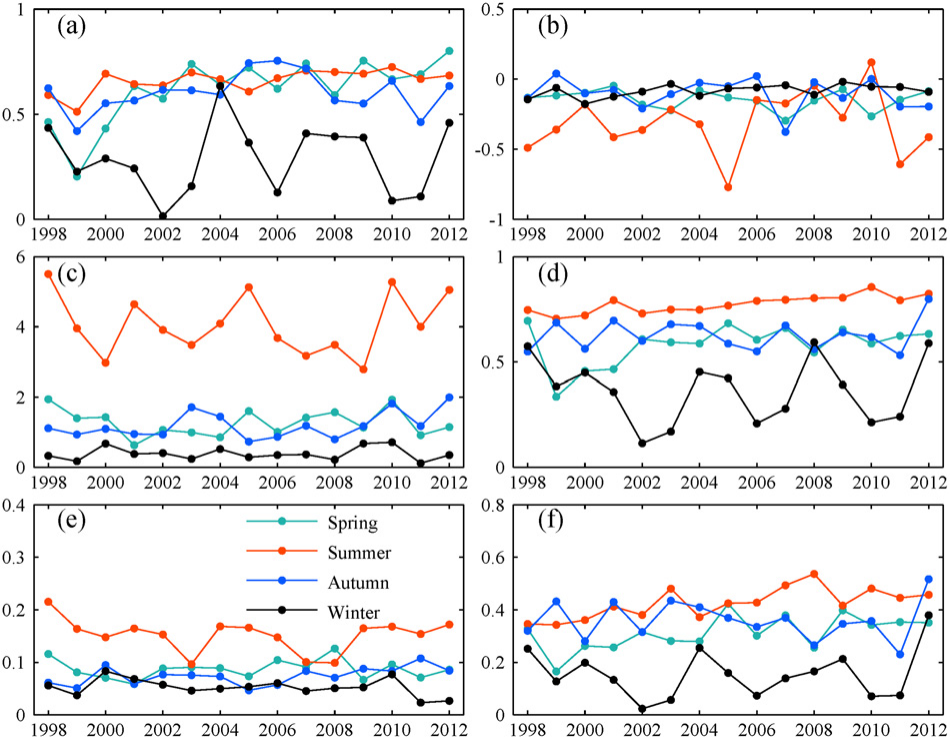
Seasonal variation of statistical indices for Liaoning province: (a) *CC*, (b) *ME*, (c) *MAE*, (d) *POD*, (e) *POFD*, (f) *ETS*. Each point represents value of accuracy index for one season in a given year. All daily data belonging to each season in one year is pooled together to calculate each index across this region, respectively.

**Fig 8:**
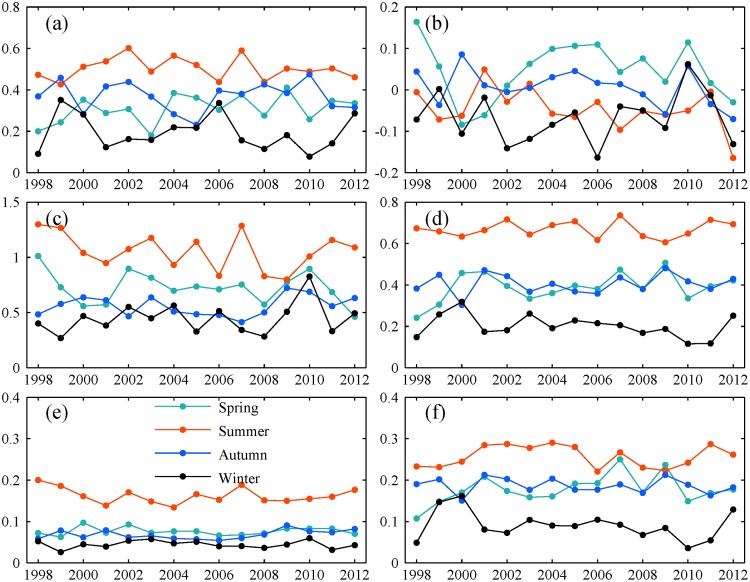
Seasonal variation of statistical indices for Xinjiang province. The legend is the same as Fig. 7.

Furthermore, two typical regions (Xinjiang and Liaoning province) located in study area are selected to gain an insight into how performance varied with season (Figs. 7 and 8). Compared to other three seasons, winter always suffered from small *CC*, low scores of *POD* and *ETS* for both regions. Because of many storms in rainy season, *ME* and *MAE* were relatively large in summer, but summer still had the best scores of the other indices. Thus, precipitation could be detected better by TMPA with a higher accuracy in summer than in winter. But it is noted that there is a distinct difference in surface condition and climate condition between winter and the other three seasons. It is characterized by low temperature and frequent snowfall events. This maybe has a negative effect on detecting precipitation by satellite. Also, the flaws in precipitation retrieval algorithms partly contribute to the worse performance in winter.

### Temporal and spatial analysis of accuracy indices

#### Temporal variation

**Fig 9:**
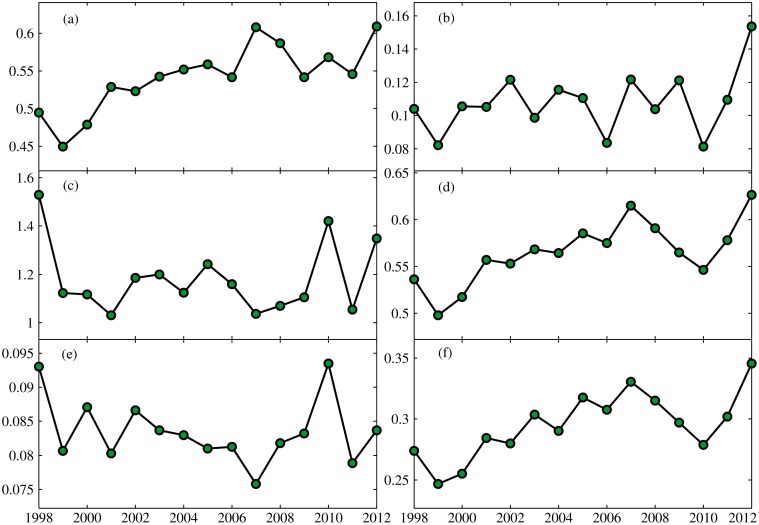
Annual variation in the six accuracy indices: (a) *CC*, (b) *ME*, (c) *MAE*, (d) *POD*, (e) *POFD*, (f) *ETS*. Each point indicates the mean of respective indice for all stations for each year.

Fig. 9 presents the annual variation of accuracy indices in the period of 1998–2012. Six accuracy indices were calculated based on daily precipitation time series for each individual station for each year, separately. Then, the average value of the respective indice for all stations for each year was used for analysis of annual variation. Overall, in terms of *CC*, *POFD*, *POD* and *ETS*, the performance of TMPA has been improved in some degree. *CC*, *POD* and *ETS* exhibited an increasing trend over time, but *POFD* had a decreasing trend during 1998–2012. It suggested that the correlation between TMPA and rain gauge became stronger, and TMPA data could be more effective in detecting precipitation events with the improved statistical scores during 1998–2012. In terms of *ME* and *MAE*, errors in TMPA fluctuate with an insignificant trend in the period of 1998–2012. The improvement in TMPA V7 makes great contribution to the changing in performance. A sophisticated Global Precipition Climatology Center full gauge analysis with improved climatology and anomaly analysis was considered for TMPA V7, which had a positive effect on the accuracy of TMPA data in predicting precipitation, especially in complex terrain [37]. More satellite observations were merged into the new version of TMPA, including 0.07° NCDC Grisat-B1 infrared data and SSMI/S, which improved the resolution and areal coverage over the infrared data (1°, 24-class histograms) used in the V6 algorithm. In addition, TMPA data benefits from the enhanced TRMM L2 PR product [44]. Judging from Fig. 9, the difference in precipitation amount between TMPA and rain gauge remained steady, but the detection of precipitation events by TMPA becomes more accurate. Usually, occurrence of precipitation events identified correctly is crucial to weather forecasting and agricultural management. The improved performance causes TMPA data to hold great promise for practical application.

**Fig 10:**
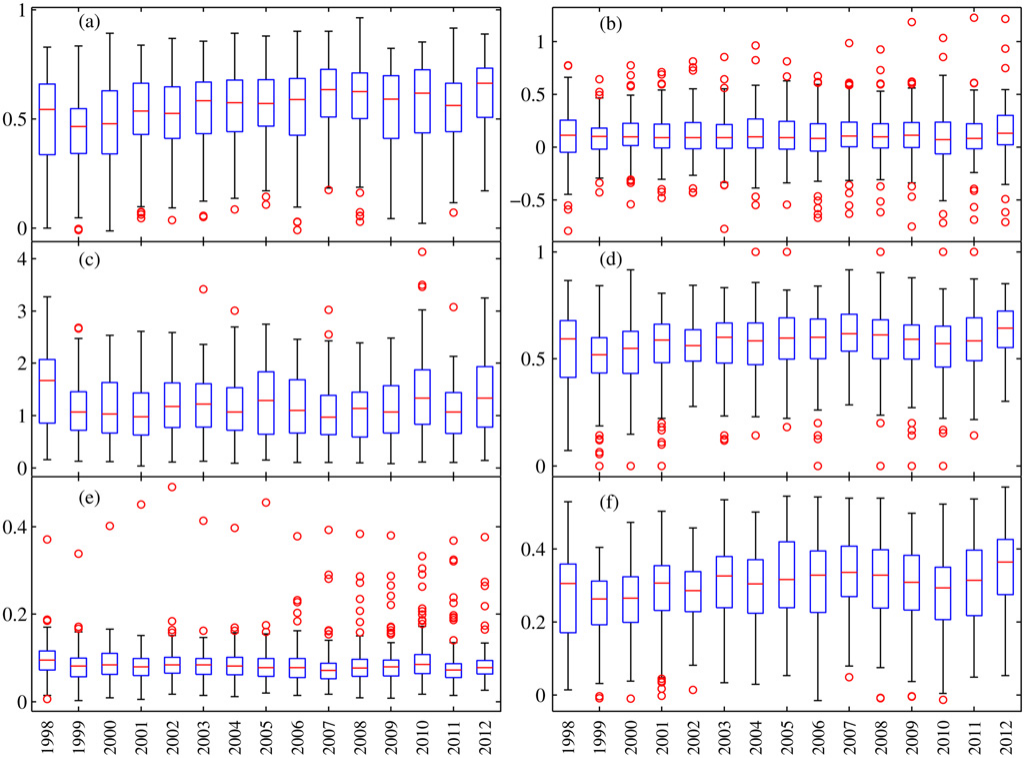
Boxplots of the six accuracy indices used in this study: (a) *CC*, (b) *ME*, (c) *MAE*, (d) *POD*, (e) *POFD*, (f) *ETS*. Each box shows the 25th and 75th percentiles of distributions of each accuracy index. The horizontal line shows the median of the distributions, and the whiskers extend out to largest and smallest values within 1.5 times the interquartile range. And the circle represents outliers.

Boxplots describe the statistical distribution of respective indices for all stations for each year (Fig. 10). The distribution of accuracy indices for each year was close to a Gaussian distribution. However, note that many so called “outliers” exist in some stations for all year, such as low *CC*, abnormal under- or overestimation of precipitation, or a low hit rate but a high detection false rate. The spatial analysis of indices discussed below provides some clear reasons for this.

#### Spatial analysis

**Fig 11:**
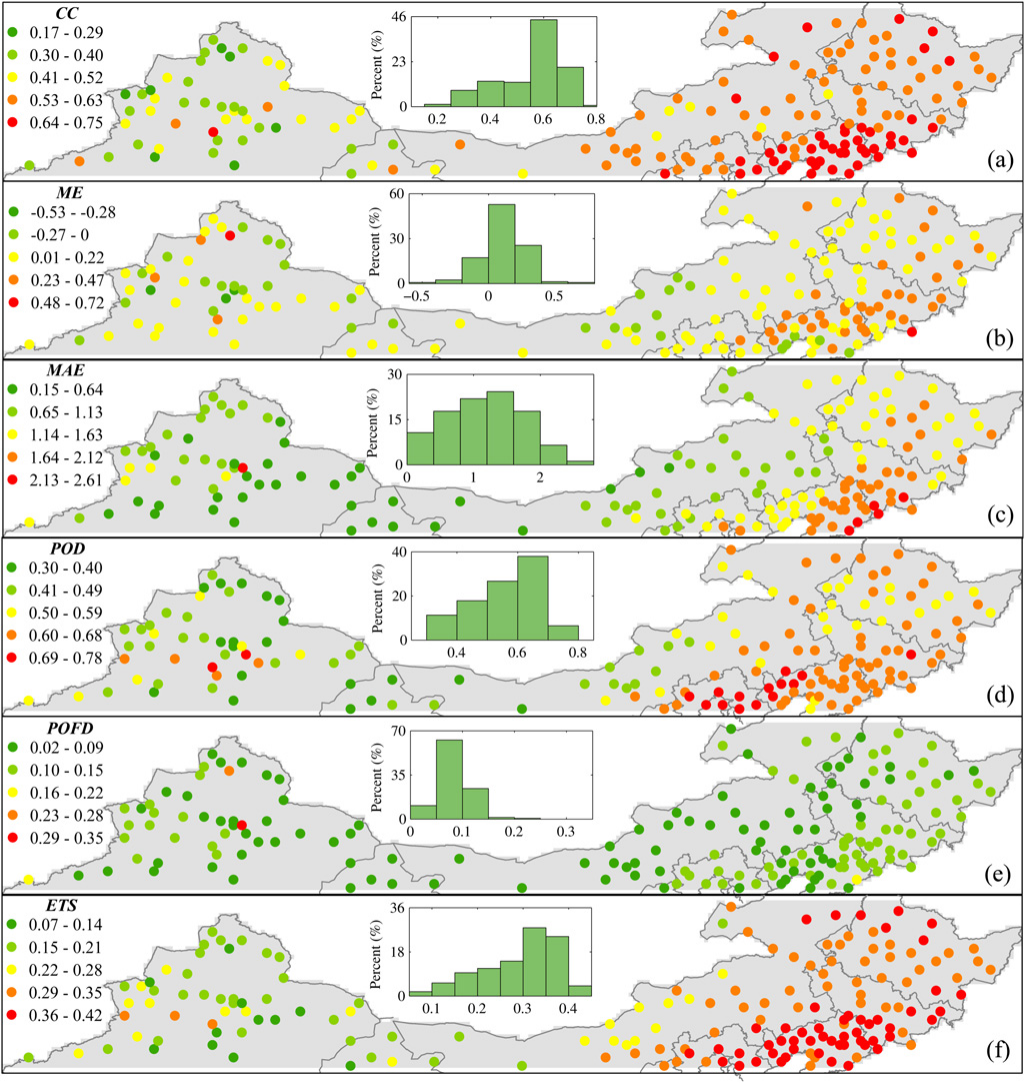
Spatial distribution of accuracy indices based on daily precipitation data during 1998–2012: (a) *CC*, (b) *ME*, (c) *MAE*, (d) *POD*, (e) *POFD*, (f) *ETS*. Each point is the center of one TMPA grid box which includes at least one rain gauge. The shaded region indicates precipitation during summer and winter.

In addition to the analysis of temporal variation, detailed spatial distribution of accuracy indices were also explored (Fig. 11). *CC* exhibited a distinct spatial pattern across the study area with an upward trend from west to east ranging from 0.17 and 0.75 (Fig. 11A). In the western part, it is an arid area characterized as scarce precipitation, high altitude and mountainous area, only a weak correlation was observed between TMPA and rain gauge. Particularly, TMPA always suffers from a low *CC* that is smaller than 0.5 in Xinjiang Province. However, the value of *CC* was improved greatly in the eastern part, especially in Liaoning Province where it was almost larger than 0.63. A histogram (see the inset in Fig. 11A) also indicated that the value of *CC* between 0.5 and 0.7 accounts for more than 70%, indicating a moderate correlation tends to predominate over this mid-high latitudes region. Generally, the value of *CC* is sensitive to range of the amount of precipitation. It is evident that there is a distinct difference in precipitation regimes between the eastern and western part. Rare big convective storms occur in the western part, and the precipitation rate is small, compared to the eastern part. Thus, this may partly result in a smaller *CC* in the western part.

Fig. 11B and C show two quantitative statistics, *ME* and *MAE*, used for evaluating the amount of precipitation. The spatial distribution of *ME* was relatively homogeneous over this region. Even though the geography of the western and eastern parts of the study area differ significantly, *ME* still show a similar pattern in both regions. Therefore, this suggests the pattern of *ME* is not dependent on geographical characteristics. Under- and overestimation of precipitation by TMPA coexist over this region (Fig. 11B), and overestimation plays a dominant role. TMPA generally tends to slightly overestimate precipitation, by less than 0.47 mm, over this region. Adequate attention should be paid to this problem when a TMPA product is used to drive climate/hydrological models. For *MAE*, there is a significant spatial pattern increasing from west to east. This trend agrees well with the spatial distribution of precipitation. The eastern part is rich of precipitation with heavy intensity. By contrast, precipitation is rare with low intensity and few occurrences in the arid western part. Due to limitation of satellite sensor discussed above, TMPA has a large possibility of giving larger errors in precipitation in the eastern part than the western part. Fortunately, the absolute error in precipitation is not large, within 2.61 mm.

In order to understand how well TMPA can detect precipitation events, spatial distribution of three categorical statistics, *POD*, *POFD* and *ETS* are illustrated in Fig. 11D-F. Compared to *POD* and *ETS*, *POFD* did not show a significant difference in spatial pattern, but had a distinct homogeneous nature over the entire study area (Fig. 11E). Apparently, *POFD* was independent of geographical characteristics, the nearly same score is over the western and eastern part. Lower *POFD* scores of less than 0.15 dominated the spatial pattern of *POFD*, in particular, *POFD* less than 0.1 could account for more than 79% among all rain gauges. This suggests that TMPA generally tends to have a low probability of false detection. Nevertheless, the other two categorical indices exhibit a similar spatial pattern with an upward trend from west to east. Visual comparison between *POD* and *POFD* (Fig. 11) suggest that TMPA usually performed better in the eastern part with larger *POD* but similar *POFD*, especially *POD* almost were greater than 0.59 around the Bohai Sea. For example, scores of *POD* were 0.67 and 0.47 averaged over Liaoning and Xinjiang province, but these two areas had the same *POFD* with 0.09. Spatially, lower *POD* scores were mainly distributed in semi-arid and arid areas in the western part (in western Inner Mongolia, to northwestern Gansu, to Xinjiang). In these areas, the intensity of precipitation is so low that satellite sensors often fail to detect it as a result of the limitation of minimal detectable signals [5]. Therefore, a decrease in correctly detecting precipitation events by TMPA resulted in lower *POD* scores. Besides, Fig. 11F shows a significant spatial pattern for *ETS*. The score of *ETS* increased from west to east, ranging from 0.07 to 0.42. Apparently, TMPA provides better scores in the eastern part than that in the western part.

### The difference in performance between TRMM covered and no TRMM covered area

**Fig 12:**
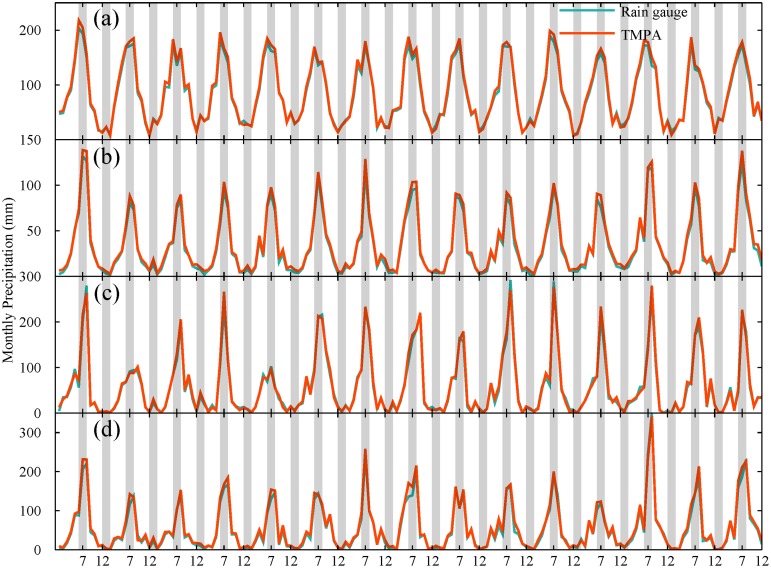
Variation of monthly precipitation from rain gauges and TMPA averaged over (a) 20-40°N region covered by TRMM, (b) 40-50°N region not covered by TRMM, (c) Shandong province and (d) Liaoning province.

**Fig 13:**
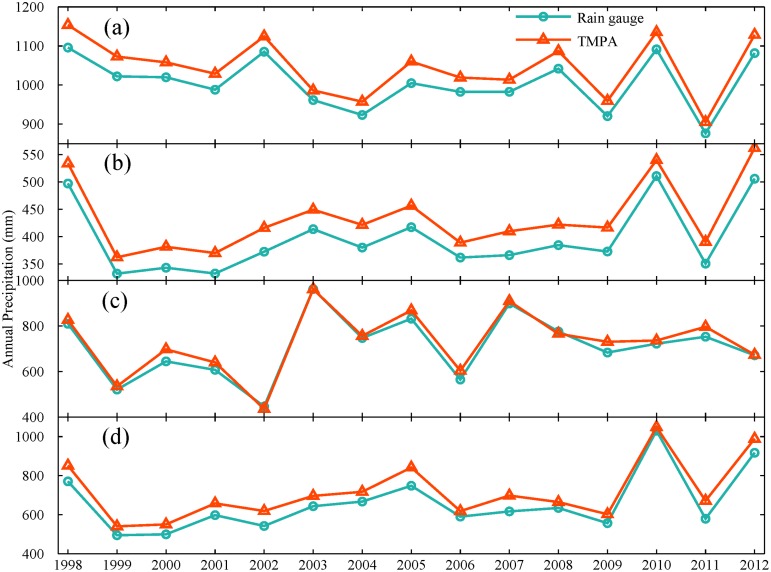
Variation of annual precipitation from rain gauges and TMPA averaged over four regions: (a) 20-40°N region covered by TRMM, (b) 40-50°N region not covered by TRMM, (c) Shandong province and (d) Liaoning province.

Furthermore, a preliminary analysis on the effect of calibration on performance is conducted over TRMM-covered (latitude between 20 and 40°N in China, hereinafter “20-40 region”) and no TRMM-covered region (40-50°N in China, hereinafter “40-50 region”). Also the other two special areas are selected to provide some detailed information: one is Shandong province located in TRMM coverage; the other is Liaoning province located in no TRMM coverage. They have similar geographical and climatic characteristics, as listed in Table 2. TMPA shows a consistent behavior for all selected regions, it can capture variation and trend in monthly and annual precipitation well (Figs. 12 and 13). There is a slight difference in monthly precipitation during rainy season. However, an apparent systematic overestimation by TMPA is shown for all four regions (Fig. 13). It is noted that the overestimation in TRMM covered area is smaller than that in non-TRMM covered area with 4.06% and 9.77% for 20-40 region and 40-50 region, respectively. Thus, the calibration for input satellite for precipitation retrieval partly contributes to the improved performance in 20-40 region covered by TRMM satellite.

Specifically, the performance of TMPA for two typical subregions (Shandong and Liaoning Province) is examined. As shown in Fig. 12, the observed monthly precipitation could be detected by TMPA with a considerable high degree of correspondence for both subregions. There is no significant difference in performance of monthly precipitation for these two areas. However, TMPA showed a distinct behavior to capture annual precipitation (Fig. 13). Though the variation and trend of annual precipitation were captured well by TMPA, TMPA overestimated more heavily the amount of annual precipitation in Liaoning than Shandong province. The result of *ME* also supported this, with 58.51 mm for Liaoning and 19.82 mm for Shandong, as listed in Table 3. It is noted that the overestimation of annual precipitation in Liaoning (8.87%) is about three times as many as that in Shandong (2.79%). Obviously, TMPA performed worse in no-TRMM covered area.

**Fig 14:**
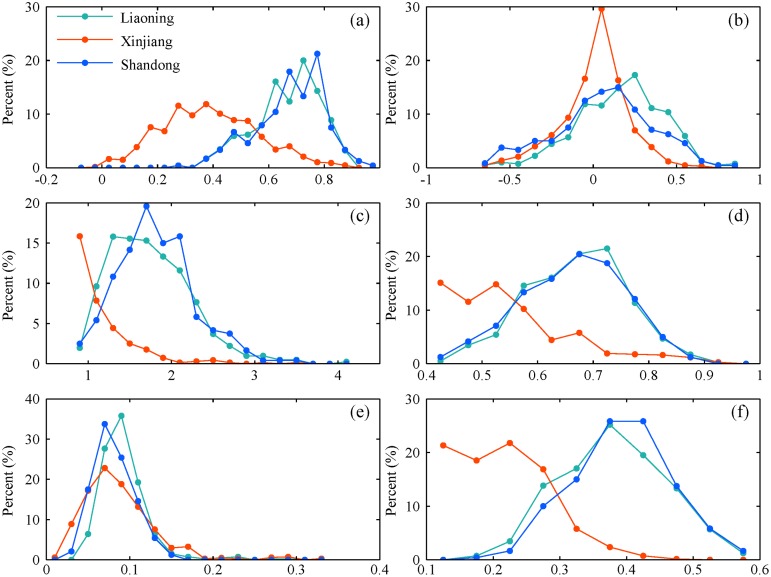
The frequency distribution of the six statistical indices for three regions during 1998–2012: (a) *CC*, (b) *ME*, (c) *MAE*, (d) *POD*, (e) *POFD*, (f) *ETS*. Each statistical index is computed for individual rain gauge for each year.

In addition, the frequency distribution of statistical indices for Liaoning and Shandong was also investigated to access TMPA’s performance in different areas (Fig. 14). Each statistical indice was calculated based on daily precipitation for every year in individual station in each region. The frequency of six indices in Liaoning is similar to that in Shandong. Usually, TMPA got a good score with a high correlation, desirable *POD* and *ETS*. By contrast, the distribution of statistical indices in Xinjiang was unique, except for *ME* and *POFD*. Generally, a poor statistical score dominated in this region, especially *CC*, *POD* and *ETS*. Thus, TMPA has a limited accuracy to detect precipitation characteristic in arid area.

## Discussion and Conclusion

TMPA has a general overestimation of precipitation amount (see in Figs. 5, 12 and 13). Previous studies in both low (Saudi Arabia [43]) and high (Laohahe basin in China [27]) latitudes regions have provided some evidence related to the characteristics of the overestimation of precipitation. Our findings support this argument and also indicate that overestimation seems to be inherent in TMPA products despite some improvements in the algorithm and use of additional data sources. It is possible that compared with rain gauge observation, this overestimation of precipitation by TMPA may be attributed to some limitations of the retrieval algorithms (e.g., no physical relationship between rain rate and bright temperature in infrared data and the effects of snow or ice on passive microwave data) and data quality. It is worth noting that it would result in some unexpected peak flows while utilizing TMPA in hydrological applications directly. Besides, in terms of *CC*, relative and absolute bias percentage, TMPA was found to have the poorest performance at daily scale. The correlation improves significantly, from 0.63 to 0.95, with an increased time scale. This trend is consistent with results reported in the Central Andes region [45], the western part of Kenya [24] and the Zambezi River Basin [46]. The use of monthly rain gauge data for bias adjustment in TMPA contributes greatly to this improvement [18]. Also, TMPA heavily overestimates precipitation at daily scale with a similar relative bias (7.48%) but had the largest absolute bias (106.54%), whereas the relative and absolute bias are 7.88% and 31.64% for monthly time series, 7.87% and 16.63% for annual time series. Clearly, TMPA could be more suitable for reproducing a reasonable precipitation time series at a larger timescale (month or year). The severe degree of overestimation in daily TMPA data might be weakened by effective bias correction with adequate sub-daily or daily observational data, which makes it possible to serve as an alternative daily precipitation data source, especially for hydrological applications and weather forecasting in ungauged basins. In addition, the result for TRMM-covered area indicates that the calibration indeed improves precipitation estimation. The overestimation of monthly and annual precipitation by TMPA in TRMM-covered region would be roughly one third of that in no TRMM-covered area.

Also, the TMPA accuracy varies in different seasons. The correlation coefficient decreases progressively from 0.66 in summer, to 0.62 in autumn, to 0.56 in spring, to the worst value of 0.33 in winter. The most heavy overestimation of precipitation occurred in winter compared to other seasons. Most likely, this can be attributed to the distinct differences in surface conditions during cold and warm season. Typically, the area in China between 40°N and 50°N has low temperatures and frequent snowfall in winter. The surface is covered with ice or snow for long time. According to precipitation retrieval algorithms, precipitation derived from microwave data relies on scattering signals over land [19]. But frozen and icy surfaces cause strong scattering, which results in estimation errors in the cold season. This type of surface makes it a tough and challenging work for satellites [21, 37]. Besides, Joyce and Arkin [47] reported that infrared retrieved estimation of precipitation was also severely affected by snow cover and cold air masses. Moreover, a good skill in detecting precipitation events also appears in summer with the best scores of *POD*, *POFD* and *ETS*, but winter still gets the worst scores. Generally, TMPA cannot provide a reasonable and reliable information regrading precipitation in winter, thus driving snowmelt runoff model with TMPA would generate unrealistic runoff and be unable to forecast spring floods especially in snow dominated regions. Considering that snow or ice has a severe impact on the accuracy of TMPA, research related to the elimination or reduction of the interference caused by noise signals should be implemented to enhance the physical relationship between effective satellite signals and precipitation estimates.

The temporal variation of accuracy indices shows that it demonstrates a mild increasing trend in performance of TMPA during 1998–2012. In fact, the orbital altitude of the TRMM satellite was boosted from 350 to 402.5 km [48] in August 2001 for prolonging its lifetime, which led to some changes in swath width and field-of-view size of the sensors. Many researchers have expressed concern about the accuracy of TMPA, but our results prove that potential impacts of the orbit boost on precipitation estimates was not as serious as expected. Great effort has been made by TMPA developers to eliminate this impact, resulting in an improved performance of TMPA.

According to spatial pattern of six indices, TMPA can perform better at capturing precipitation events in the eastern part (such as Liaoning Province) than in the western part (especially in arid area, such as Xinjiang Province). Accuracy indices shows a significant spatial pattern increasing from west to east as followings: *MAE* (0.35–2.56 mm), *POD* (0.22–0.85) and *ETS* (0.04–0.39). The spatial distribution of both *POFD* and *ME* were relatively homogeneous, and were independent of geographic features. Judging from *ME*, a slight overestimation of precipitation (<0.47 mm) by TMPA prevails over this mid-high latitudes region. In semi-arid and arid areas, TMPA does not show desirable performance, and this would directly hinder its application to climatic and hydrological research, especially in ungauged regions. In the future, more accurate satellite data are needed, they can be incorporated into processing a more accurate precipitation product that can enhance the poor results of TMPA in semi-arid and arid areas. Overall, TMPA is quite qualified for humid area. Consequently, TMPA are not suitable for analyzing the characteristics of rainfall and could not serve as an alternative source of precipitation data to drive climatic or hydrological models in arid areas over mid-high latitudes regions. The undesirable input data would result in misleading streamflow simulations.
